# Protruded duodenal tumor arising from Santorini’s duct of the pancreas: a rare case of intraductal papillary mucinous neoplasm mimicking a duodenal polypoid tumor

**DOI:** 10.1186/s12876-020-01449-y

**Published:** 2020-09-16

**Authors:** Haruna Komatsubara, Hiroyuki Kato, Daisuke Noguchi, Kazuyuki Gyoten, Aoi Hayasaki, Yusuke Iizawa, Takehiro Fujii, Akihiro Tanemura, Yasuhiro Murata, Naohisa Kuriyama, Masashi Kishiwada, Hiroyuki Sakurai, Shugo Mizuno

**Affiliations:** 1 Department of Hepatobiliary Pancreatic and Transplant Surgery, Graduate School of Medicine, Mie University, 2-174 Edobashi, Tsu, Mie, 514-0001 Japan; 2 Department of Hepatobiliary Pancreatic and Transplant Surgery, Mie University, Tsu, Japan

**Keywords:** Duodenal tumor, Santorini’s duct, Intraductal papillary mucinous neoplasm

## Abstract

**Background:**

We experienced a rare case of intraductal papillary mucinous neoplasm arising from Santorini’s duct (SD) forming a tumor protruding into the duodenum .

**Case presentation:**

A 71-year-old woman was incidentally diagnosed with a 3 cm type Isp polypoid tumor in the second portion of the duodenum at another hospital. Enhanced CT and endoscopic ultrasound revealed that the origin of this protruding tumor was arising from SD and that the tumor mimicked a pedunculated duodenal tumor. Our preoperative diagnosis was a malignant pancreatic tumor arising from SD with invasion into the duodenum. She underwent a subtotal stomach-preserving pancreaticoduodenectomy, and the resected specimen showed a 25 mm tumor protruding into the duodenum with a villous surface. The pathological findings revealed that the tumor was intraductal papillary mucinous adenoma (IPMA) arising from SD.

**Conclusions:**

To the best of our knowledge, this is the first case of IPMA protruding into the duodenal lumen from SD, although most of the tumors arising from SD have been reported to be malignant.

## Background

Duodenal neoplasms are rare entities accounting for less than 1% of all gastrointestinal tumors and are characterized by their location in the duodenum and proximity to the ampulla [1]. On the other hand, duodenal polyps are found in 1.5–4.6% of routine esophagogastroduodenoscopy (EGD) procedures and 7% are reported to be diagnosed as adenoma [2]. The current treatment options for duodenal tumors include open surgical resection and endoscopic techniques such as endoscopic mucosal resection and submucosal dissection, but a precise treatment strategy has yet to be established.

Furthermore, the increased detection rate of pancreatic cysts due to the improvements and increases in the number of imaging studies has led to a surge in interest in intraductal papillary mucinous neoplasms (IPMNs) of the pancreas. IPMNs are usually classified into three types based on imaging studies and/or histology based on the origin of the tumor whether it arises from the main pancreatic duct (MPD), branches of the main ductal system, or both of them. According to these classifications, the treatment strategy was varies from conservative observation to pancreatic resection.

Regarding the location of pancreatic IPMNs, Santorini’s duct (SD) is rarely the origin of IPMNs [3, 4]. Moreover, there has been no report in which an IPMN originating from SD protruded from the minor papilla, mimicking a duodenal polypoid tumor. We herein report a rare case of an IPMN arising from SD, forming a duodenal pedunculated tumor. To the best of our knowledge, this is the first case report of a protruded duodenal polypoid tumor.

## Case presentation

**Fig. 1:**
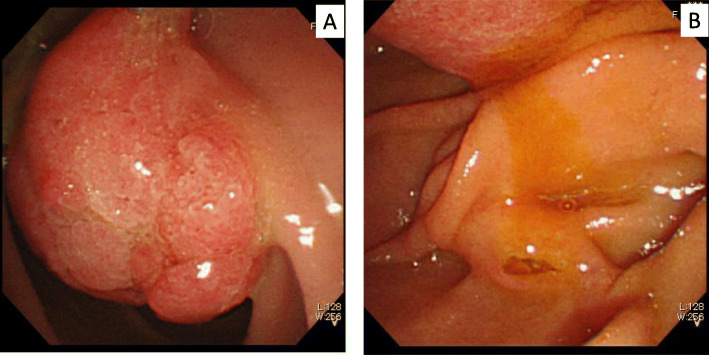
Gastroduodenal endoscopy findings. A polypoid tumor is located at the second portion of the duodenum (**a**). The orifice of the major duodenal papilla is mildly enlarged, and slight mucin production is seen (**b**)

A 71-year-old woman was incidentally diagnosed with diabetes mellitus with an elevated HbA1c, and a multimodal assessment was conducted to search for its cause. Her medical history included acute pancreatitis of unknown etiology and laparoscopic cholecystectomy for gallstones. Abdominal computed tomography (CT) in another hospital showed a mass lesion in the second portion of the duodenum and dilation of the main pancreatic duct (MPD). Gastroduodenal endoscopy incidentally showed a 3 cm type Isp polypoid tumor in the second portion of the duodenum (Fig. 1a) and slight mucin production from the major papilla (Fig. 1b). Thus, she was referred to our hospital for further evaluation. The endoscopic tumor biopsy showed papillary-tubular neoplasm with low grade dysplasia, but she was not diagnosed with a malignancy based on the degree of cellular atypia. On admission, her serum amylase level was 203 U/L, HbA1c was 12.4% and other biochemical data were within the normal range. Regarding tumor markers, her level of carbohydrate antigen (CA) 19–9 was elevated by 60.2 U/ml, carcinoembryonic antigen (CEA) and duke pancreatic monoclonal antigen type 2 (DUPAN2), which is a serum marker for pancreatic cancer that when elevated, suggest the presence of malignancy, were within a normal range though.

**Fig. 2:**
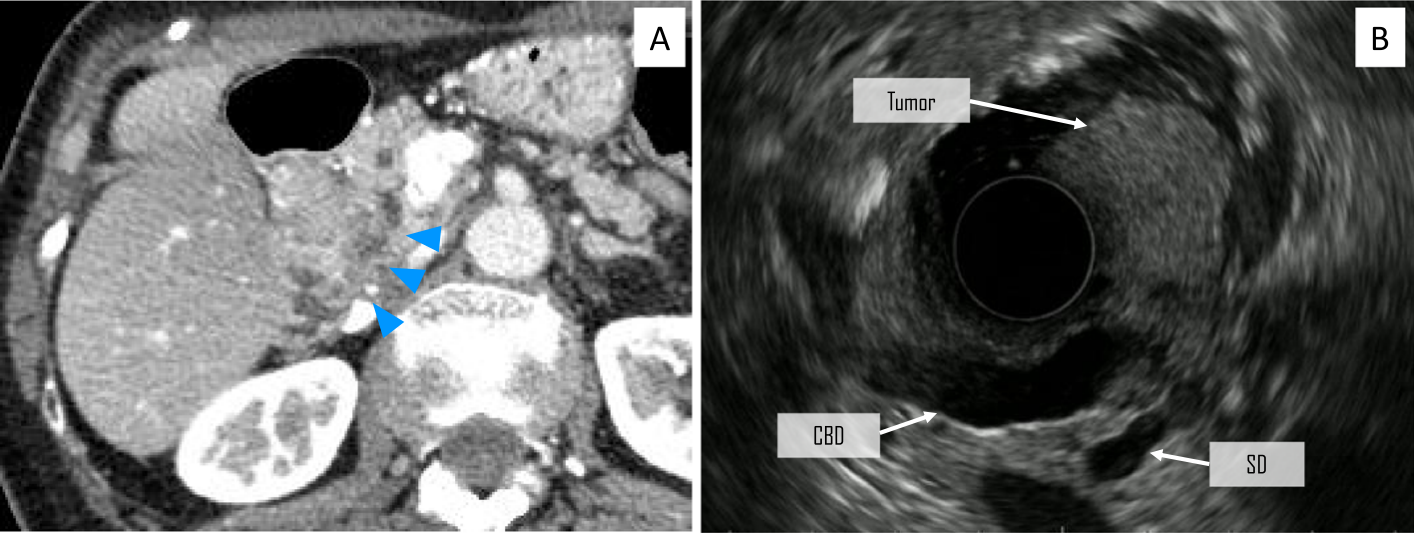
Preoperative imaging studies. Contrast-enhanced abdominal CT imaging demonstrates an enhanced mass in the second part of the duodenum, which is adjacent to the dilated SD (arrowhead) (**a**). Radial EUS showed that the tumor protruded into the duodenum from the SD (**b**). CT: computed tomography, EUS: endoscopic ultrasound, SD: Santorini’s duct, CBD: common bile duct

**Fig. 3:**
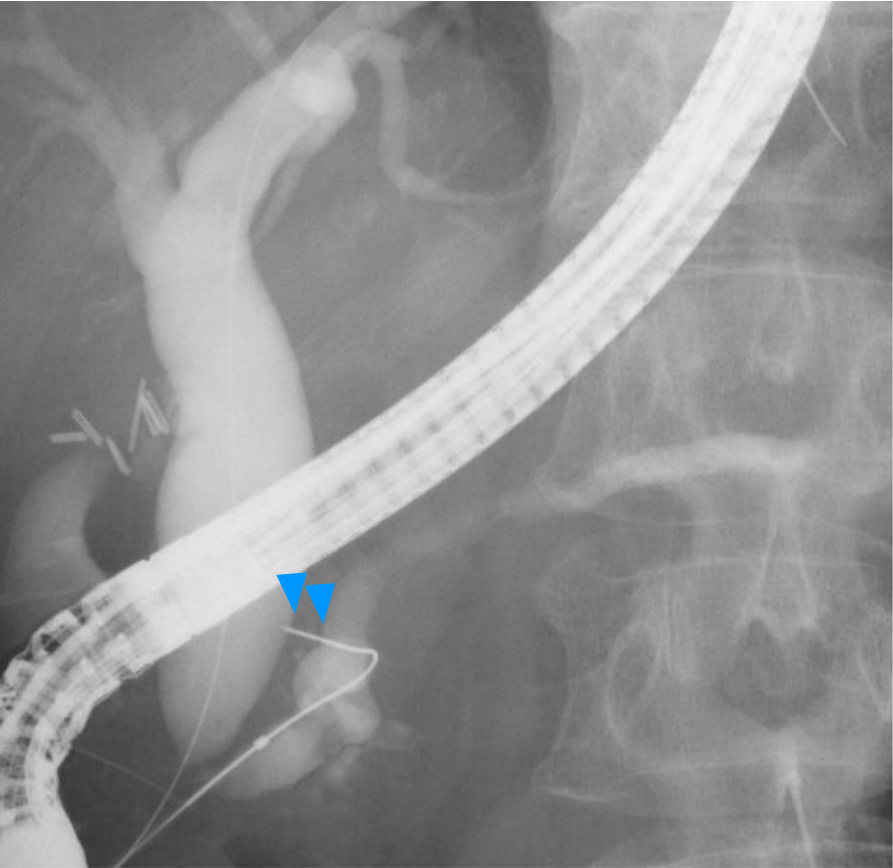
ERCP delineated the entire main pancreatic duct, but not SD, which might be due to the mucin production from the tumor (arrowheads). ERCP: endoscopic retrograde cholangiopancreatography, SD: Santorini’s duct

Thin-slice enhanced abdominal CT imaging in our hospital demonstrated an enhanced mass (approximately 25 mm in size) in the second part of the duodenum, which is adjacent to the dilated SD. The dilated SD also had an enhanced nodule, suggesting an intraductal tumor (Fig. 2a). Endoscopic ultrasound (EUS) revealed a dilated SD and irregular mucosal thickness in its branches. In the region of SD, an ill-defined low echoic area that continuously extended to a pedunculated tumor at the orifice of the minor papilla was discovered (Fig. 2b). Endoscopic retrograde cholangiopancreatography delineated the entire main pancreatic duct, but SD was not well delineated as shown in Fig. 3. The preoperative diagnosis was a malignant pancreatic tumor arising from SD with invasion into the duodenal lumen. She underwent subtotal stomach-preserving pancreaticoduodenectomy and her postoperative course was uneventful.

**Fig. 4:**
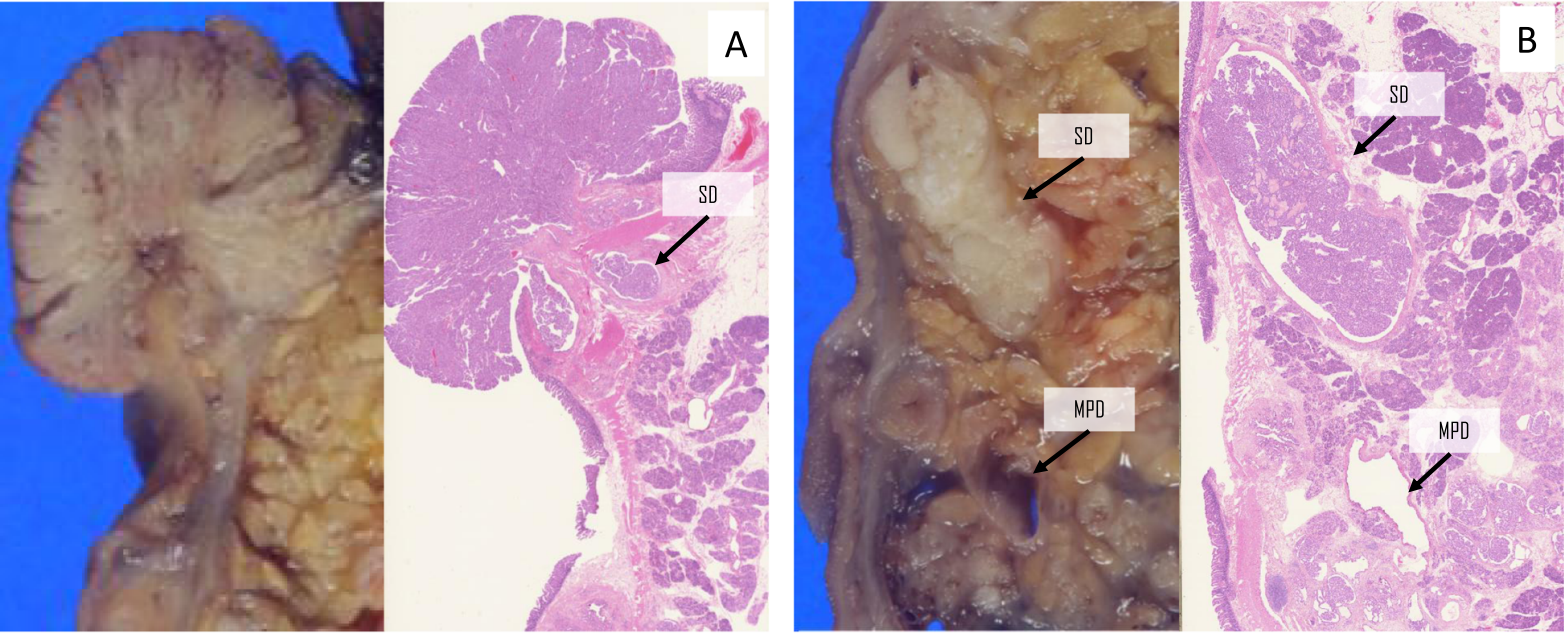
Macroscopic findings of the resected specimen. The resected specimen shows the tumor protruding into the duodenum, which was adjacent to the dilated SD (**a**), and a grossly visible intraductal tumor within SD and its branches (**b**). MPD: main pancreatic duct, SD: Santorini’s duct

**Fig. 5:**
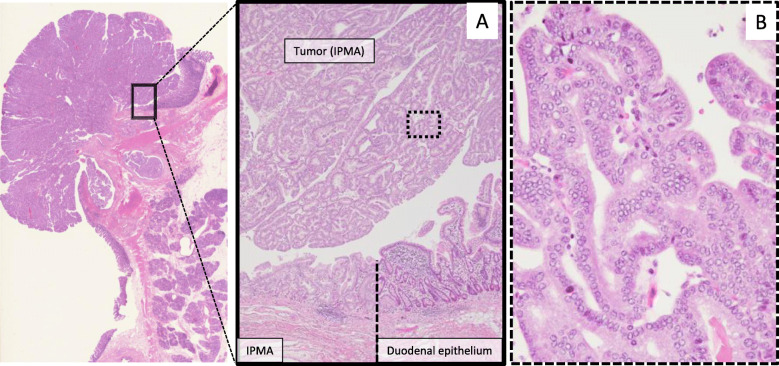
Microscopic findings of a duodenal tumor. The transition region between the IPMA (left and upper side) and normal duodenal mucosa (right side) (**a**). This intraductal tumor has intermediate level nuclear atypia (**b**) and was diagnosed as an IPMA with intermediate dysplasia. IPMA: intraductal papillary mucinous adenoma

Macroscopically, the resected specimen showed a 25 mm duodenal projection with a villous surface (Fig. 4a), which was the same composition as the grossly visible, cylindrically dilated SD (Fig. 4b). Microscopically, the tumors were characterized by the intraductal proliferation of columnar mucin-producing cells, which form papillae ranging from the microscopic fold to grossly visible projections. This tumor had moderate nuclear atypia, and stromal invasion was not observed. In this case, the primary focus was recognized to be arising from SD, although the tumor involved not only SD but also its branches. These findings herein led to the diagnosis of an IPMA of the gastric type with intermediate dysplasia (low-grade IPMN in the 5th edition of the WHO tumor classification) originating from SD and its branches (Fig. 5a and b).

## Discussion and conclusions

In the present case, discrimination between a duodenal polypoid tumor and IPMN was difficult, because a type I papillary polypoid tumor was seen in the second portion of duodenum by EDG, mimicking a duodenal polypoid tumor. However, EUS revealed that the low echogenicity area continuously extended to a pedunculated tumor at the orifice of the minor papilla, allowing us to make a precise diagnosis preoperatively.

In the differential diagnosis of this unusual IPMN, superficial non-ampullary duodenal tumors (SNADET), which are defined as lesions that are limited to the duodenal mucosa or submucosa, including adenoma and/or adenocarcinoma was regarded as one of the possible diagnoses. SNADETs are grossly classified according to the Paris endoscopic classification, and the gross morphology is classified based on endoscopic findings and divided into pedunculated (Ip), sessile (Is), semipedunculated (Isp), superficial elevated (IIa), completely flat (IIb) or superficial shallow or depressed types (IIc) [5]. To treat SNADETs, surgical resection such as limited local resection, endoscopic mucosal resection (EMR) and endoscopic submucosal dissection (ESD) are indicated according to the tumor size [6]. In this case, this polyp seemed to be an Isp SNADET, but the tumor size was larger than 20 mm; thus, endoscopic resection was contraindicated, even if this tumor was assumed to be a SNADET.

To discrimination between anomalous IPMN and SNADET in our case, EUS was very useful for identifying that this pedunculated polyp was protruding from SD of the pancreas, allowing us to perform the proper surgical treatment for this rare type of tumor.

IPMNs arising in SD or its branches are quite rare, and only 11 cases have been reported in the English literature as shown in Table 1 [7–12]. Among them, 9 cases were considered carcinoma, and only 2 cases were diagnosed as adenoma. With regard to the duodenal tumor formation, only our case shows a tumor arising from SD protruding into the duodenal lumen. Although Miyake et al. [10] reported a patient with tumor formation at the minor papilla of the duodenum, the tumor was a malignant IPMN arising from SD that invaded to the duodenal wall directly, which is different from our case. To the best of our knowledge, this is the first case of an IPMA protruding into the duodenal lumen from SD.

**Table 1:** Summary of cases of intraductal papillary mucinous neoplasm arising from SD in the literature

Case		Year	Age	Gender	Primary lesion	Protrusion to duodenal lumen	Operative procedure	Pathological diagnosis	Prognosis (months)
1	Saito 9)	1989	74	F	SD	–	inoperable	adenocarcinoma with liver metastasis	4 (dead)
2	Miyake 10)	2004	67	M	SD	–	SSPPD	Adenocarcinoma*	ND
3	Kanazumi 11)	2004	71	M	SD	–	PPPD	adenocarcinoma	13 (alive)
4	Hirano 2)	2005	60	M	SD	–	PD	adenocarcinoma	ND
5	Hirano 2)	2005	73	F	SD	–	DPPHR	adenocarcinoma	ND
6	Hirano 2)	2005	66	M	Branch of SD	–	PPPD	adenocarcinoma	ND
7	Hirano 2)	2005	46	F	SD	–	PRPD	adenocarcinoma	ND
8	Akashi12)	2013	78	F	Branch of SD	–	PD	adenocarcinoma with cholangiocarcinoma	59 (dead for other illness)
9	Abe 7)	1998	65	M	Branch of SD	–	PD	adenoma with carcinoma of cystic duct	15 (alive)
10	Hirano 8)	2005	63	F	Branch of SD	–	DPPHR	adenoma	ND
11	our case		71	F	SD	+ (25 mm)	SSPPD	adenoma	14 (alive)

*SD* Santorini’s duct, *SSPPD* Subtotal stomach-preserving pancreaticoduodenectomy, *PD* Pancreaticoduodenectomy, *DPPHR* Duodenum-preserving pancreas head resection, *PPPD* Pylorus-preserving pancreaticoduodenectomy, *PRPD* Pylorus-resecting pancreatoduodenectomy, *ND* not described

* Showing direct duodenal invasion

Regarding the prognosis of IPMNs derived from SD, Hirano et al. [7] reported that this type of tumor showed an unfavorable prognosis with compared to that of the tumors arising from the MPD. However, the precise prognosis of these tumors has yet to be elucidated because of the small number of patients, insufficient follow-up period, and variations in tumor differentiation from adenoma to adenocarcinoma. Therefore, more cases need to be accumulated to elucidate the clinicopathological characteristics and prognosis of these tumors.

We experienced a rare case of an IPMA protruding into the duodenal lumen from SD, although most of the tumors arising from SD have been reported to be malignant.

## Data Availability

All data generated or analyzed during this study are included in this published article.

## Abbreviations

EGD: Esophagogastroduodenoscopy
IPMN: Intraductal papillary mucinous neoplasm
MPD: Main pancreatic duct
CT: Computed tomography
CA19–9: Carbohydrate antigen 19–9
CEA: Carcinoembryonic antigen
DUPAN2: Duke pancreatic monoclonal antigen type 2
EUS: Endoscopic ultrasound
SNADET: Superficial non-ampullary duodenal tumors
SD: Santorini’s duct
CBD: Common bile duct
IPMA: Intraductal papillary mucinous adenoma
SSPPD: Subtotal stomach-preserving pancreaticoduodenectomy
PD: Pancreaticoduodenectomy
DPPHR: Duodenum-preserving pancreas head resection
PPPD: Pylorus-preserving pancreaticoduodenectomy
PRPD: Pylorus-resecting pancreatoduodenectomy
ND: Not described
